# On the Fluid Secretions in Cholera

**Published:** 1849-08

**Authors:** 


*On the Fluid Secretion in Cholera.* By M. Mialhe.—M. Andral established in a paper read to the Academy of Sciences, in 1847, that "the white fluid which fills the intestines of cholera patients is not actually a part of the blood, as has often been maintained, for neither albumen nor fibrine can be found in it; but that it is formed by mucus, secreted suddenly, and in large quantities, and by that very fact greatly altered in quality." By analyses made during the present epidemic, I find that the ejected fluid contains neither fibrine nor albumen, but yields, by certain chemical re-agents, a plentiful precipitate, which precipitate is *albuminose.* This latter substance is, as I have lately shown, the final product of the digestion of albuminous articles of food; just as glucose is the final product of the digestion of amylaceous matters. Albuminose has characters in common with albumen; both are soluble in water, and insoluble in alcohol, both give a precipitate by the addition of salts of leads, mercury, and silver; by chlorine and tannin. But albuminose, when isolated, does *not* coagulate by heat or acids, not even by nitric acid. These two latter properties will at once distinguish albuminose from albumen. Now the cholera dejections coagulate neither by heat nor nitric acid, which fact points to the absence of albumen; they give an abundant precipitate on the addition of alcohol, the salts of lead, mercury and silver, of chlorine, tannin, &c., which precipitate is albuminose. The next question is, how this albuminose comes to be secreted by the economy! Albuminose is not formed at once; it can only take its origin in the albuminoid portions of the blood, and in the living tissues. The albuminous and fibrinous parts of the blood undergo, by the effects of cholera, a transformation analogous to that which would result from the digestion of these same principles in the stomach under the influence of pepsine. Muscles are liable to the same metamorphosis, and are thus exposed to a sort of interstitial absorption.

The albuminose, thus formed, instead of ministering to the nutrition of patient, as is the case in prolonged low diet, is, on the contrary, immediately driven from the system, both by the gastro-intestinal mucous membrane and the skin, to which latter it gives a very characteristic abnormal viscosity. The first albuminose secreted is furnished by the whole mass of the blood, and the muscles undergo their molecular transformation only secondarily! This muscular absorption explains very satisfactorily the rapid phenomena of prostration and loss of flesh so remarkable in cholera patients. But if the albuminose arising from the blood found no difficulty in passing from the torrent of the circulation into the digestive cavities, it is far different with the albuminose which is derived from muscular interstitial absorption, for this second variety must, before oozing npon the surface of mucous membranes, pass through the capillary system, in order to enter the circu'ation. Hence we shall have an arrest of circulation, and consequent upon it, cyanosis, algidity, and asphyxia, at the moment of the interstitial absorption giving rise to considerab,e tumidity, (eight or ten times the original volume,) which the fibrine undergoes in order to dissolve and be metamorphosed into albuminose. Such are the material causes which, according to my views, produced the hitherto unexplained phenomena of this dreadful disease. Thus it appears that the dejections of cholera contain an enormous proportion of albuminose, and this albuminose can only be derived from the organic elements of the system. In searching for the cause of this extraordinary perturbation, one would be led to admit a fermentiferous principle, which gives rise to phenomena entirely analogous to those of the digestion of albuminoid articles of food in the stomach. When the economy appropriates to itself the albuminous principles ingested into the body, it must subject them to a constitutive molecular decomposition, which effaces from them all traces of organization. The system then assimilates them, and gives them a new organization, which is the origin of albumen, fibrine and cruor. Now it must be noticed, on the other hand, that these principles, thus newly formed, cannot leave the economy without having been completely disorganized. This is exactly what takes place in cholera; a disease in which the presence of albuminose in the secretion is an evident proof of a disorganizing transformation. For were it otherwise, we should see, not albuminose, but albumen, fibrine, and cruor escaping from the vessels, as by a kind of expression, through the intestinal mucous membrane, as most authors have hitherto believed. Starting from this hypothesis, it looks as if we could arrest or suspend the abnormal phenomena of cholera in the same way as certain fomentations are stopped. But all the substances calculated to produce this effect are unfortunately too active or poisonous; as the salts of lead, copper, mercury, and silver, mineral acids, &c. They would, in fact, be more dangerous than the disease itself. Tannin (and all the medicines which act in virtue of this substance,) would seem rather desirable, owing to its faint action on the mucous membrane; but in spite of the favorable results obtained by Dr. Graff, of Berlin, it should not be forgotten that Tannin, at the same time as it acts upon the fermentiferous substances, acts also upon the albuminoid principles, and that its coagulating powers must, in large doses, be rather dangerous. It seems extremely probable, that the different prophylactic means which have been extolled in cholera, some being violent excitants, as mustard, pepper, cajeput oil, naptha, creosote, the sesquichloride of carbon, solutions of potassa, soda, ammonia, and their various salts; others, sedative or anæsthetic agents, as opium and its preparations, Indian hemp, ether, and chloroform, act by producing in the economy, the first, a powerful reaction; the others, a stupefaction, which does not allow of the morbid disorganization. I regret, after having investigated the nature and the causes of the choleric affection, to have no especial method of cure to bring forward. I may, perhaps, conclude by stating, that every one of the above mentioned means may become all-powerful in the hands of an enlightened practitioner.

A circumstance of a very curious nature was lately noticed on making the post-mortem examination of a man who had died of cholera at the

Hospital St. Antoine. The *bladder* was found to contain a small quantity of the pecular fluid forming the alvine dejections.—*Lancet, April*, 1849, *from L' Union Med.*

